# Editorial: Women in insomnia

**DOI:** 10.3389/frsle.2025.1683978

**Published:** 2025-09-03

**Authors:** Darlynn M. Rojo-Wissar, Jessica M. Meers, Patricia L. Haynes

**Affiliations:** ^1^Department of Psychiatry and Human Behavior, Alpert Medical School of Brown University, Providence, RI, United States; ^2^Bradley/Hasbro Children's Research Center, E.P. Bradley Hospital, East Providence, RI, United States; ^3^Department of Medicine, Baylor College of Medicine, Houston, TX, United States; ^4^Department of Health Promotion Sciences, The University of Arizona Mel and Enid Zuckerman College of Public Health, Tucson, AZ, United States

**Keywords:** insomnia, women, sleep health, social context, gender disparities

Women in public health, medicine, and social sciences make up a large and growing proportion of today's scientists studying insomnia. However, significant gaps in gender equity remain. Women scientists in academia publish less, are cited less, hold fewer grants, and remain underrepresented at the most senior levels of their field. Women in academia also face major pay disparities and biases arising from long-standing gender norms that serve as barriers to research productivity and advancement. To fight these structural inequalities and encourage equitable representation, we present a Research Topic of highly innovative and impactful research from women in insomnia, which highlights three core themes.

The first theme that emerged was the interest in examining the biological and social constructs unique to women that impact sleep health. Benge et al. summarized the current literature on sleep across the unique timepoints in a woman's biological life, including pregnancy, menopause, and in relation to gynecological conditions. They also explored the role of social factors on sleep health in women from racially and ethnically minoritized backgrounds and LGBTQ+ populations. The authors described how each of these contextual factors increases the risk of a range of sleep concerns, with a particular emphasis on the role of stress and affective conditions as precipitators of insomnia.

Webster et al. examined a similar contextual variable that tends to impact women at higher rates than men: being a caregiver, particularly in those of advanced age. Interestingly, their findings provide a degree of optimism, as caregiver status did not increase the likelihood of experiencing insomnia symptoms, thereby suggesting that there may be some protective value to being a caregiver at home. While caregiving may buffer against insomnia in some contexts, Bennaroch and Shochat found that the combination of shift work, stress, and eveningness poses a substantial risk to insomnia and mental health in female nurses.

Several other manuscripts in this Research Topic expanded on the importance of social context for sleep. Giorgio Cosenzo et al. explored how Latinx men and women experience insomnia through social processes such as stress, control, and support, and found that Latina women specifically attribute their insomnia to their social identities as women, mothers, and caretakers. The mini review by Lara et al. showed that sleep disturbances can also be deeply tied to social environments during *childhood*, summarizing the effects of early-life adversity on sleep disturbances, and positing subsequent impairments in brain and language development.

Altier et al. connected gratitude, a socially embedded emotion, to better sleep via greater health self-efficacy, more adaptive health behaviors, and lower psychological distress in primary care patients. Together, these three manuscripts demonstrate that the social context, through stress, support, identity and resilience, profoundly affects sleep health. The findings suggest that greater consideration of the social context in interventions targeting insomnia, vs. focusing solely on individual behaviors, may boost treatment effectiveness.

Finally, three randomized controlled trials in this Research Topic highlighted the diversity of intervention modalities used by clinical researchers in the field. In their randomized controlled trial of post-9/11 veterans with PTSD, Bristol et al. highlighted the interpersonal benefits of service dog partnerships on sleep quality and sleep disturbances, as compared to a waitlist control group. A key contribution of this work was the identification of how human-dog partnerships may alleviate the fear of sleep, underscoring the role that these animals play in providing comfort with minimal nighttime disruption.

Cromer et al. demonstrated that individual-level Cognitive Behavioral Therapy (CBT) for nightmares led to significant improvements in children after only five sessions. The successful delivery of this intervention through a healthcare Zoom platform underscores its accessibility, offering an effective approach to overcoming barriers in behavioral sleep treatment access among children and their families.

Using a robust sham condition, Huskey et al. found minimal direct effects of repetitive transcranial magnetic stimulation (rTMS) on insomnia-related cognitive impairment. However, their research suggests a potential neurophysiological mechanism for mitigating the cognitive effects of sleep inertia through earlier timing and enhanced consolidation of slow-wave sleep (SWS) and REM sleep.

These contributions offer valuable insights into novel interventions that also deepen the understanding of intervention mechanisms at the social, individual, and physiological levels. Taken together, these studies showcase the innovative contributions of women researchers to insomnia research and emphasize the need to further refine and expand opportunities for women scientists in this field.

